# Editorial: Mechano-Calcium, Mechano-Electric, and Mechano-Metabolic Feedback Loops: Contribution to the Myocardial Contraction in Health and Diseases

**DOI:** 10.3389/fphys.2021.676826

**Published:** 2021-04-06

**Authors:** Rémi Peyronnet, Olga Solovyova, Gentaro Iribe, Leonid B. Katsnelson

**Affiliations:** ^1^Institute for Experimental Cardiovascular Medicine, University Heart Center Freiburg Bad Krozingen, Freiburg, Germany; ^2^Medical Center and Faculty of Medicine, University of Freiburg, Freiburg, Germany; ^3^Institute of Immunology and Physiology, Russian Academy of Sciences, Ekaterinburg, Russia; ^4^Department of Physiology, Asahikawa Medical University, Asahikawa, Japan

**Keywords:** myocardium contraction, electromechanical coupling, mechano-electric coupling, mechano-mechanical feedback, calcium transient, action potential, frank-starling law

Sensing of and adaptation to mechanical forces in the heart are allowed in part by Mechano-Electric Coupling (MEC), including mechano-electric, -calcium, and -mechanical feedback mechanisms interrelating with each other. Mechanosensitivity of healthy and diseased hearts has been a focus of research interests for decades (Lab, 1982, 1996, 1999; Kohl et al., 1999; Kohl and Sachs, 2001; Takahashi et al., 2013). Many mechanisms underlying MEC are still unclear. The works presented in this Research Topic involve state-of-the-art experimental approaches, computational modeling and focused reviews, aimed at shedding light on some specific aspects of MEC which can be subdivided as follow.

## MEC and Cardiac Remodeling

Cardiac metabolic, mechanical, electrical, and structural remodeling allows the heart to adapt its function to the demand. Such plasticity of the cardiac tissue and cells in response to hemodynamic loads is comprehensively reviewed by Pitoulis and Terraciano. Two more articles presented in the Research Topic also relate to remodeling (Abu-Khousa et al., Seo et al.).

## Frank-Starling Law and Underlying Mechanisms

Three articles deal with “Force-Length” and “Pressure-Volume” relationships in single cardiomyocytes, multicellular preparations, and whole ventricle. Using a mathematical model of cardiac excitation-contraction, Guidry et al. compared end-systolic force-length relationships (ESFLR) simulated in isometric and work-loop contractions. These simulations show that the isometric ESFLR curve lies significantly higher than its work-loop counterpart, being in good agreement with the data of numerous experiments on muscle preparations and the whole heart. The authors attribute these results to the mechanosensitive kinetics of Ca^2+^ -TnC, while further analysis seems necessary.

The same team of researchers discovered close correlations between the stress-length and heat-stress relations in both isometric and work-loop contractions over wide ranges of preloads and afterloads in experiments on rat trabeculae (Tran et al.). Both relations changed in codirectional manner depending on mechanical loading. These data demonstrate tight interdependence between mechano-mechanical and mechano-energetic feedback loops.

To predict how perturbations of molecular level mechanisms in myocardium will scale up to modulate system-level properties, Campbell et al. developed a multiscale hemodynamic mathematical model using a single contractile element to drive a single hemisphere ventricle that pumps blood around a closed circulation. They found that the End-Systolic Pressure Volume Relationship was the most sensitive to small changes in molecular-level parameters compared to other system-level properties. The results suggested that a subtle deficit in nearly any aspect of sarcomere-level function could compromise contractility.

## Location Matters for MEC

Not only ventricle mechanosensitivity, but also that of other heart regions has been studied in the articles presented in this Topic. The pulmonary veins (PV) have been targeted as the source of the atrial arrhythmogenic activity. Egorov et al. investigated stretch-induced MEC to the development of arrhythmogenic ectopic beats from pulmonary veins. They found that pathological stretch facilitates the development of arrhythmogenic ectopic activity induced by high concentrations of adrenaline in pulmonary veins resulting in intra-PV conduction, frequent episodes of unstable reentrant activity, and atrial extra beats. These findings highlight an arrhythmogenic impact of pulmonary vein stretch in the development of atrial arrhythmias under elevated autonomic tone which could play a critical role in patients with elevated blood pressure.

Comparison of the chronotropic response of the isolated sinoatrial node (SAN) to stretch in rabbit and mouse allowed better understanding of interplay between structural, mechanical, and electrophysiological properties of the SAN as highlighted by MacDonald et al. Importantly, pharmacological interventions aimed to prolong mouse SAN action potential plateau duration resulted in stable positive chronotropic response similar to that of rabbit and other species.

Khokhlova et al. showed that preload might be a source for mechano-calcium-electric modulation in myocyte electrophysiological and contractile function across the wall. They experimentally studied the effect of stretch on the force characteristics of rat subendocardial and subepicardial single ventricular cardiomyocytes and analyzed the results using mathematical models. They concluded that greater myofilament length-dependent activation in subendocardial cells compared to subepicardial cells via MEC led to the reduction in the transmural differences in action potential duration at a higher preload.

## Calcium, T-System, and Transient Receptor Potential (TRP) Channels in MEC

Simulations carried out in one-dimensional computational model of electro-mechanics in rabbit ventricular cardiomyocyte with mechanically non-uniform sarcomeres (Timmermann and McCulloch) showed that myocyte stretch sequences may contribute significantly to the delayed after-depolarizations due to mechanically-triggered calcium waves in myocytes overloaded with calcium. In 1D multi-cellular chain models where such myocytes were coupled via gap junctions, the mechanically-induced waves contributed to synchronizing arrhythmogenic calcium waves and afterdepolarizations (*ibidem*).

This work seems to be in line with earlier studies, although those were focused on various mechanisms underlying the studied phenomena: one dealt with contribution of mechano-calcium feedbacks to arrhythmogenesis in cardiomyocytes overloaded with Ca^2+^ analyzed in a mathematical model (Sulman et al., 2008); the second reported on calcium waves triggered by heterogeneous contractions (Ter Keurs et al., 2005).

Lookin and Protsenko investigated how the extent of length-dependent activation (LDA) in cardiac cells may relate to the diastolic level of calcium and calcium-transient peak rest and/or activator calcium in rat cardiomyocytes. They have found that the extent of LDA is not determined by actual peak systolic tension but regulated by the Ca^2+^-transient peak magnitude of activator calcium and the kinetics of the Ca^2+^-transient decline. Their findings suggest that complex mechanisms of mechano-calcium feedback are involved with LDA.

Ahmad et al. describe the location and effects of transient receptor potential canonical 6 (TRPC6) channels in neonatal rat ventricular myocytes (NRVM) induced with an adenoviral vector to express enhanced-green fluorescent protein (eGFP) or TRPC6-eGFP. Induced NRVM exhibited increased sarcoplasmic reticulum calcium load compared to eGFP cells. These experimental findings were supported by ionic model simulations. Obtained results deepen the understanding of the processes underlying the stretch-induced activation of TRPC6 channels. Overexpression of TRPC6 channels may be involved in development of cardiac hypertrophy. This opens a new target for pharmacological therapies based on TRPC6 inhibitors.

Positive force-frequency relationship (FFR) is a characteristic of the healthy human heart. In chronically failing hearts the FFR becomes flat or negative. Reasons for this are not clearly established. Abu-Khousa et al. investigated whether remodeling of the transverse tubular system (t-system) could explain the alteration of the FFR. They show using human cardiac tissue slices that the remodeling of the t-system can predict FFR alterations.

Complementary to the analysis of the t-system alteration on the FFR, the impact of transverse tubule loss was next assessed on the slow force response. The latter and the β-adrenergic activation are shown to critically require transverse tubules (Power et al.).

Ischemic tissue exhibits altered mechanics and is more prone to arrhythmias. A better understanding of the mechanisms underlying such pro-arrhythmic substrate is the focus of the work from Cameron et al.. Ischemia is shown to enhance both stretch-induced increase of Ca^2+^ spark rate and the associated ROS production. Such mechanism may contribute to the generation of Ca^2+^-induced arrhythmias.

## Stretch-Induced Intracellular Signaling

Molecular adaptations to mechanical overload during hypertrophy and heart failure is a subject of the review presented by Seo et al.. In response to overload, cardiac tissue adaptation processes are multifaceted and involve a large number of molecules. It has been suggested that angiotensin II type 1 receptor (AT_1_R) and apelin receptor (APJ) are primary upstream actors with indirect mechano-sensing capacities contributing to both Ca^2+^-dependent and Ca^2+^-independent inotropic effects. Seo et al. review the impact of AT_1_R and APJ signaling pathway activation by ligand *vs*. mechanical stimuli on inotropy and cardiac hypertrophy. The relevance of these receptors as therapeutical targets in the context of heart failure is also discussed.

## Tools and Methods for MEC Investigation

Articles presented in the Research Topic Collection involve and describe various methodical approaches relevant in the area under consideration.

In particular, Darkow et al. present a cell-selective pharmacological tool to study cardiac mechano-sensors. Such tools are important to recognize various mechanisms underlying MEC. All cells in the heart sense their mechanical environment and adapt to it. To do so, mechano-sensors such as stretch-activated channels are believed to be important players. Modulating their activity in a selective manner has always been a target and a challenge. Lectin A may offer an original way to sensitize TREK-1 but not Piezo1 to stretch. Interestingly, this curvature-inducing protein binds only to non-myocytes in the heart and not to cardiomyocytes.

A further noteworthy aspect of the impact of mechanical conditions on contractility and electrical activity of the cardiomyocytes is disclosed in the review dealing with preconditioning of cultured cells (Peretz et al.). It turns out that elimination of contraction during culturing preserves important MEC properties of cultured myocytes, in particular, cultured pacemaker, and atrial cells.

Elimination of contraction was implemented also by Kappadan et al. in Langendorff experiments with whole hearts. Particular effects of contractions on myocardial electrical activity are still a matter of debate. Different studies demonstrated either shortening or lengthening, or no response of action potentials in isolated cells or myocardial tissue to the mechanical perturbations. Kappadan et al. utilized optical mapping techniques to compare ventricular electrophysiology in beating *vs*. contraction-inhibited isolated rabbit hearts. They observed shorter action potential durations in contracting hearts. The results do not allow one to distinguish pure effects of contraction from possible effects of mild hypoxia in contracting hearts during Langendorff-perfusion (Garrot et al., 2017). Further studies might elucidate the matter.

## Energetics

Last but not least, two studies focus on mechano-energetic coupling. In addition to the above-cited article by Tran et al. dealing with the energetics equivalent of the Cardiac FLR, there is the article by Barclay and Loiselle studying quantitative determination of the thermodynamic efficiency of ATP hydrolysis. This work was initiated by a mismatch between ATP yield per mole of glucose inferred from thermal studies compared to that seen in mitochondria oxidation [see *e.g.*, Rich (2003) or Salway (2004)]. The authors tried to probe this discrepancy using a thermodynamically constrained algebraic model of cardiac mechano-energetics. They conclude that the mismatch is due to different techniques between myothermic and mitochondrial experiments. Future improvement of technology in the field may reconcile them and further the understanding of cardiac mechano-energetics.

Considering long-term perspectives, it would be interesting to study other manifestations of the mechano-energetic coupling, *e.g.*, investigating whether different mechanical loading conditions of the cardiomyocyte are associated with differences in energy consumption. Are there intracellular mechanisms that might allow maintaining the necessary level of ATP in the cytosol under any mechanical loading, and if so, what are these mechanisms? Perhaps, this topic will be explored in the future along with a host of other subjects from the exciting field of cardiac mechano-sensitivity and adaptation. The interesting results reported and discussed within this Research Topic will hopefully stimulate future studies in this area.

## Author Contributions

All authors contributed to the first draft of the manuscript, manuscript revision, and read and approved the submitted version.

## Conflict of Interest

The authors declare that the research was conducted in the absence of any commercial or financial relationships that could be construed as a potential conflict of interest.
